# Efficient molecular dynamics using geodesic integration and solvent–solute splitting

**DOI:** 10.1098/rspa.2016.0138

**Published:** 2016-05

**Authors:** Benedict Leimkuhler, Charles Matthews

**Affiliations:** 1 School of Mathematics and Maxwell Institute of Mathematical Sciences, University of Edinburgh, James Clerk Maxwell Building, Peter Guthrie Tait Road, Edinburgh EH9 3FD, UK; 2 Department of Statistics, University of Chicago, 5734 S. University Avenue, Chicago, IL 60637, USA

**Keywords:** constrained molecular dynamics, biomolecular simulation, high-dimensional sampling, Langevin dynamics, Brownian dynamics, stochastic differential equations

## Abstract

We present an approach to Langevin dynamics in the presence of holonomic constraints based on decomposition of the system into components representing geodesic flow, constrained impulse and constrained diffusion. We show that a particular ordering of the components results in an integrator that is an order of magnitude more accurate for configurational averages than existing alternatives. Moreover, by combining the geodesic integration method with a solvent–solute force splitting, we demonstrate that stepsizes of at least 8 fs can be used for solvated biomolecules with high sampling accuracy and without substantially altering diffusion rates, approximately increasing by a factor of two the efficiency of molecular dynamics sampling for such systems. The methods described in this article are easily implemented using the standard apparatus of modern simulation codes.

## 1. Introduction

Molecular dynamics (MD) is a widely used and powerful tool for studying molecular systems with extensive applications to the simulation of macromolecules both for fundamental biology/biochemistry [1,2] and, increasingly, for medical applications [3]. In MD simulation of complex systems, the most important restriction (ignoring issues of force field quality) is the size of the timestep that can be used to accurately compute trajectories. The goal of simulation is typically to unlock behaviours that occur on timescales of microseconds or more, for example state-to-state protein conformational dynamics [4] such as partial folds of proteins [5], but the use of typical MD methods limits the timestep to a few femtoseconds.

In recent decades, with the explosion in the use of MD, there has appeared a wide variety of integration algorithms for molecular dynamics simulation, with the typical goal being to sample the canonical ensemble defined by constant temperature, particle number and system volume. The two principal types of tools that are in widespread use for increasing the timestep in molecular sampling are multiple timestepping [6,7] and the use of holonomic constraints [8–10]. Caution is needed when applying the former in MD because (i) many schemes exhibit significant resonance artefact [11,12] that substantially limits their viability for practical applications and (ii) methods to tame the resonances may end up severely corrupting the calculation of dynamical properties such as transition and diffusion rates.

The method of constraints consists of introducing algebraic relations, for example to freeze selected bond lengths [9,10] and/or bond angles [13]. It is commonly stated that the use of constraints can enable an increase in the simulation timestep for organic molecules in detailed solvent to between 2 and 4 fs, a substantial improvement on the 1–2 fs typically used for fully flexible models. It is worth mentioning that a doubling of the timestep may mean halving the computational budget for a laboratory that relies largely on such MD simulations, so the cost impacts (or, conversely, the accessible range of simulation) owing to increasing the timestep are considerable. The general wisdom regarding the stepsize threshold for biomolecular dynamics, with constrained *stochastic* dynamics, still places the limit at around 4 fs [14]. Efforts to go beyond this have largely involved more complicated computations (e.g. Hessian matrices and/or partial normal modes, and schemes for mollification of resonance artefacts) [15–18], often limiting their efficiency (or scalability on parallel architectures) or their suitability for implementation in major molecular modelling software. Typical methods are paired with special techniques to compute the long-range electrostatic forces, such as particle mesh Ewald summation. Some extreme multiple timestepping proposals can use ‘outer’ stepsizes (in a multiple timestepping context) of more than 100 fs [19–21], but without any consideration of the dynamical approximation.

There is no general principle for constructing integrators for constrained stochastic systems such as Langevin dynamics. Extending an idea of Leimkuhler and Patrick [22] for deterministic dynamics on a Riemannian manifold, we propose to view the splitting of a constrained MD problem as a collection of separate constrained problems and to solve or approximate each constrained flow. In practice, the constraints are implemented using one or more steps of the SHAKE method [8] (or its RATTLE variant [23]). As particular examples, we obtain geodesic versions of a popular splitting due to Bussi and Parinello [24] and our own ‘BAOAB’ integrator [25,26]. We find that the accuracy and stability advantages of the geodesic BAOAB scheme are so dramatic (across a wide range of stepsizes) as to render many popular alternatives uncompetitive. In the case of a biomolecular model in detailed solvent, the use of very large stepsizes leads to instabilities because of unconstrained solute modes. In this case, we show that combining the geodesic BAOAB integrator with a multiple timestepping scheme based on solvent–solute force splitting, the stepsize for biomolecules can be increased to 8 or even 9 fs without significantly compromising the resolution of long timescale dynamics or sampling accuracy and with only modest additional cost per timestep. This approach is conceptually simple and straightforward to implement using existing, off-the-peg, integration components present in most MD packages (and without the need for Hessian matrices, knowledge of specific collective variables, normal mode analyses or other system-dependent analysis).

## 2. Numerical methods for constrained Langevin dynamics

The analysis of MD stability barriers has largely focussed on deterministic MD, although some studies have emphasized the use of Langevin dynamics [15,17,24,27]. The incorporation of stochastic perturbations of the force field, combined with friction in such a way as to preserve the fluctuation–dissipation relation, dramatically changes the nature of the molecular dynamics problem. Resonances present in the deterministic system are destroyed and the ergodic nature of stochastic dynamics improves the accuracy and reliability of numerical methods, even in the long time limit.

The mathematical formulation of constrained Langevin dynamics [28] builds on the standard theory of constrained Hamiltonian systems (which may be derived starting from the constrained Euler–Lagrange equations). We write the constrained Langevin MD system in an overdetermined form

2.1 $$\begin{array}{rl} \frac{d}{dt}q & ={\text{M}}^{-1}p, \end{array}$$

2.2 $$\begin{array}{rl} \frac{d}{dt}p & =F-\gamma p+\sqrt{2k_{B}T\gamma }{\text{M}}^{1/2}\eta (t)-\sum_{i=1}^{m}\lambda_{i}\nabla g_{i}(q), \end{array}$$

2.3 $$\begin{array}{rl} 0 & =g_{j}(q),\;j=1,2,\ldots ,m \end{array}$$

2.4 $$\begin{array}{rl} \text{and}\;0 & =\nabla g_{j}{\left(q\right)}^{T}{\text{M}}^{-1}p,\;j=1,2,\ldots ,m \end{array}$$

where **M**=diag(*m*_1_,*m*_2_,…,*m*_*N*_), with *m*_*i*_ the mass associated to the *i*th degree of freedom, ***F***=−∇*U*(***q***) is the vector of total force (with $U:R^{N}\to R$ the potential energy function), *γ* represents a prescribed friction coefficient (or collision rate) and ***η***(*t*) is a vector-valued, stationary, zero-mean Gaussian process whose components satisfy

$$\langle \eta_{i}(t)\eta_{j}(t^{\prime })\rangle =\delta_{ij}\delta (t-t^{\prime }),$$

where *δ*_*ij*_ is the Kronecker delta and *δ*(⋅) is the Dirac delta. The variable {*g*_*j*_} are smooth functions that define the constraint relationships, *λ*_*i*_ are Lagrange multipliers that are chosen to maintain the constraints, *m*>0 is the number of constraints, and *k*_*B*_*T*>0 represents the temperature scaled by Boltzmann's constant. The vector (***q***,***p*** ) represents a phase space point (position and momentum vectors), restricted to the configuration manifold $M=\{q\;|\;g_{j}(q)=0,j=1,2,\ldots ,m\}$ and cotangent space $T^{*}M=\{p\;|\;\nabla g_{j}{\left(q\right)}^{T}{\text{M}}^{-1}p=0,j=1,2,\ldots ,m\}$, respectively. We assume that the number of degrees of freedom *N* to be large, making direct sampling methods (e.g. Monte Carlo methods) infeasible or inefficient.

The co-tangency constraints (2.4) are redundant, because they are automatically satisfied along solutions that lie in the configuration manifold, but it is useful to include them here for later reference. In the case of constraints, the target canonical (NVT) probability density may be written using the Dirac delta function [28–30]

$$\rho_{\beta }^{g}(q,p\;)=Z^{-1}\exp(-\beta H(q,p\;))\prod_{i=1}^{m}\delta [g_{i}(q)]\prod_{i=1}^{m}\delta [\nabla g_{i}{\left(q\right)}^{T}{\text{M}}^{-1}p\;],$$

where *β*=(*k*_*B*_*T*)^−1^ and *Z* is a suitable normalization constant so that the total integral of $\rho_{\beta }^{g}$ is one. Both the equations of motion and the probability measure can alternatively be written in an intrinsic form or in a system of local coordinates, but we favour the cartesian form for simplicity. In what follows we assume that the constraints are non-degenerate, meaning that the Jacobian matrix of the constraints is of full rank at every point, and also that the stochastic constrained system is ergodic.

The best existing methods for unconstrained Langevin dynamics are based on a splitting and composition technique [24,25,31–33] that builds directly on the procedures used to construct symplectic integrators for Hamiltonian dynamics [34,35]. The common approach is to break the stochastic vector field into parts by writing it as an additive decomposition, with each part assumed to be solvable (in the distributional sense). For example, the BAOAB method [25] uses a splitting into three parts, where ‘B’ stands for a ‘kick’ using the force evaluated at the current position, ‘A’ represents a linear drift (along the direction of the current velocity) and ‘O’ is an exact (in the sense of the associated time-evolved probability distribution initiated from the point distribution at the initial conditions) solve of the Ornstein–Uhlenbeck stochastic differential equations associated to each component:

$$\begin{array}{rl} {\dot{q}}_{i} & =0,\\ {\dot{p}}_{i} & =-\gamma p_{i}+\sqrt{2k_{B}T\gamma m_{i}}\eta_{i}(t). \end{array}$$

The primary issue for Langevin discretization in the case of molecular dynamics is the effective perturbation of the invariant distribution induced by the numerical method, i.e. the sampling bias due to finite timestep. Although it is difficult to calculate this perturbation, it is often possible to compare, in various asymptotic limits, the perturbations and thus to select optimal methods for approximation of equilibrium averages [25,36]. In addition, it is possible to analyse special cases by direct calculation, including the harmonic model problem or perturbations thereof. Of particular interest in the biomolecular dynamics setting are results which demonstrate that stepsizes of up to 2.7 fs may be used safely for fully flexible, unconstrained (detailed solvent) models without degradation of configurational sampling accuracy [26] if the BAOAB splitting is used. This observation is likely the consequence of two facts: (i) BAOAB is exact for configurational sampling in the case of harmonic oscillators and, moreover, (ii) BAOAB has much smaller errors than alternative splitting for mildly anharmonic perturbations of harmonic oscillators [37]. It is typically assumed that the stepsize in a Verlet molecular dynamics simulation of a fully flexible biological model must be restricted to about 1 fs to have sufficient accuracy for chemical studies, where ‘accuracy’ is usually taken in terms of the drift in energy in long simulations. The increased stepsize usable for the stochastic MD method is not due to a change in the stability threshold but is a direct consequence of the specific choice of splitting scheme and the change of benchmark to statistical NVT configurational sampling from energy conservation. Note also that averages of kinetic energy and momentum-dependent observables computed using the BAOAB scheme are not as accurate as position-dependent averages—the scheme is effectively optimized for configurational sampling; however, we hasten to add that the method is convergent for all sensible kinetic quantities and so it is possible, for example, to calculate autocorrelation functions and thus diffusion rates, as we demonstrate in §7.

Two of the most popular constrained dynamics schemes in the literature are SHAKE and RATTLE. The SHAKE method of Ryckaert *et al.* [8] constrains the position variables to the constraint manifold *g*(***q***)=0 via oblique projection typically taken to be along the direction orthogonal to the manifold at the previous timestep. In contrast, the ‘RATTLE’ method [23] incorporates an additional orthogonal projection of the momenta to the cotangent space at a given point. For a deterministic system, the latter consists of the following sequence of calculations using a timestep *δt*:

$$\begin{array}{rl} q^{n+1} & =q^{n}+\delta t{\text{M}}^{-1}p^{n+1/2},\\ p^{n+1/2} & =p^{n}+\frac{\delta t}{2}F(q^{n}\;)-\frac{\delta t}{2}\sum_{i=1}^{m}\lambda_{i}^{n}\;\nabla g_{i}(q^{n}),\\ 0 & =g_{j}(q^{n+1}),\;j=1,2,\ldots ,m\\ p^{n+1} & =p^{n+1/2}+\frac{\delta t}{2}F(q^{n+1})-\frac{\delta t}{2}\sum_{i=1}^{m}\mu_{i}^{n+1}\nabla g_{i}(q^{n+1}),\\ \mathrm{and}\;0 & =\nabla g_{j}{\left(q^{n+1}\right)}^{T}{\text{M}}^{-1}p^{n+1},\;j=1,2,\ldots ,m \end{array}$$

A second set of multipliers $\left\{\mu_{j}^{n}\right\}$ must be computed in order to maintain the tangency conditions. RATTLE can be viewed as half a kick in the momenta, followed by oblique projection to the configuration manifold, then half a kick in the momenta and an orthogonal projection to the cotangent space (RATTLE projection). It has been shown previously that RATTLE and SHAKE are mathematically conjugate, i.e. formally equivalent from step to step under a change of variables, and both are symplectic [38].

In the case of multiple constraints, an iterative procedure must typically be used to solve the non-linear system to implement SHAKE projection. The method of Ciccotti *et al.* [8] uses iteration over the equations (in the manner of Gauss–Seidel iteration) performing a single Newton step on each successive constraint. An alternative is to perform a Newton iteration leading to the need to solve a system of linear equations at each step [14,39]. For the RATTLE projection, in the case of multiple constraints, a similar linear system must be solved. When the constraints result in a system of decoupled planar rigid bodies, the SETTLE method [40] is an exact solution of the constraints.

**Figure 1.**
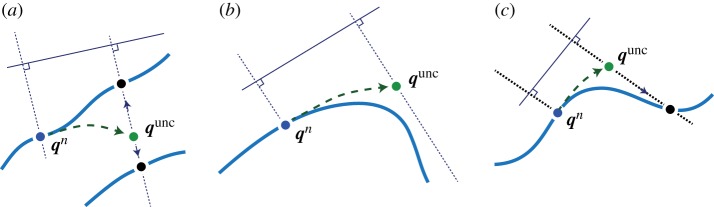
Illustration of the limitations of SHAKE projection. In SHAKE, an unconstrained step is taken from a point ***q***^ *n*^ on the constraint manifold using the applied forces resulting in ***q***^ *unc*^, then a projection back to the manifold is applied along the normal direction to the constraint manifold obtained at the start of the step. (*a*) The SHAKE projection may not be unique, and with a large timestep, even the ‘nearest’ solution might arise from a different part of the constraint manifold. (*b*) There may be no solution at all. (*c*) Even when there is a unique solution, significant error may arise from the oblique projection. (Online version in colour.)

For constrained Langevin dynamics, typical methods are constructed by interleaving unconstrained Langevin steps with projections, usually implemented via SHAKE or RATTLE steps [28,41]. In building large timestep methods, the step-project approach could, in some cases, lead to the introduction of large intermediate errors. Although the constraints are intended to be satisfied at the end of the step, there are potential problems: (i) the step may fail as there may be no solution (or more than one solution) consistent with the projection direction and (ii) even if the step succeeds, significant error may have been introduced by the procedure, due to the oblique projection (figure 1).

## 3. The geodesic integration algorithm

We now describe an integration scheme for stochastic MD which (a) preserves the constraints and (b) is symplectic in the deterministic case (*γ*=0). Although SHAKE and RATTLE are often proposed as constrained analogues of the Verlet method, we argue that the alternative approach described in this section is a more direct and geometrically coherent constrained analogue. For the deterministic case, the geodesic integrator presented here is a special case of the *Riemannian manifold symplectic integrator* [22] (specifically the method described in the comment at the end of §4.2 of that article). We combine the scheme with efficient constraint solvers, stochastic perturbation and, later in this article, a further splitting of the force field, in order to develop an effective method for biomolecular modelling.

The idea of the geodesic integrator is straightforward. If we split the Hamiltonian as *H*=*H*_1_+*H*_2_, then it is desirable to solve each part, exactly preserving the constraints and tangency constraints. Splitting Hamiltonian dynamics in the natural way into kinetic and potential terms, as in the Verlet method, and introducing Lagrange multipliers to maintain the constraints, results in a pair of systems of the form

$$\begin{array}{rl} A^{g}: & \left\{ \begin{array}{rl} & \frac{d}{dt}q={\text{M}}^{-1}p,\\ & \frac{d}{dt}p=-\sum_{i=1}^{m}\lambda_{i}\nabla g_{i}(q),\\ & 0=g_{j}(q),\;j=1,2,\ldots ,m\\ & 0=\nabla g_{j}{\left(q\right)}^{T}{\text{M}}^{-1}p,\;j=1,2,\ldots ,m \end{array}\right.\\ & B^{g}:\left\{ \begin{array}{rl} & \frac{d}{dt}q=0,\\ & \frac{d}{dt}p=-\nabla U(q)-\sum_{i=1}^{m}\mu_{i}\nabla g_{i}(q),\\ & 0=g_{j}(q),\;j=1,2,\ldots ,m\\ & 0=\nabla g_{j}{\left(q\right)}^{T}{\text{M}}^{-1}p,\;j=1,2,\ldots ,m \end{array}\right. \end{array}$$

We see that, for the kinetic part ($A^{g}$), the co-tangency constraint is redundant, but for the system in the part defined by the potential energy ($B^{g}$), the configuration constraint is redundant (since the positions are fixed), whereas the co-tangency constraint will still ensure that the changes in the momentum vector are restricted to the cotangent space. The Lagrange multipliers have been given separate names (*λ*, *μ*) to distinguish their separate roles in satisfying configurational and co-tangency conditions, respectively.

The solution for part $B^{g}$ is easily obtained. It can be viewed as a linear projection of the unconstrained kick imparted by the forces into the cotangent space. However, the solution of the $A^{g}$ part is a geodesic curve (an unforced motion on the constraint surface) and so requires the solution of a non-linear system of ODEs. A ‘geodesic integrator’ is thus obtained by solving the $A^{g}$ and $B^{g}$ parts separately and then composing the flows.

When the constraints have a simple structure, such as planar rigid bodies (e.g. water), one may compute their geodesic flow exactly. Indeed this is exactly the framework for which the SETTLE method [40] has been developed and one finds this algorithm implemented in many production codes. Accurate methods for propagating more general rigid bodies are also available and used in gas state simulations [42]. An alternative Langevin dynamics method for constrained systems of this type (i.e. which can be decomposed as a system of independent rigid bodies coupled only through forces) has been proposed by Davidchack *et al.* [43], again taking advantage of the quaternion representation to describe the constraint manifold. However, our interest is ultimately in systems of a more general type for which it is typically impossible to solve the $A^{g}$ part exactly. Nonetheless, from an efficiency perspective, we see that the geodesic integrator requires only the numerical solution of a non-linear flow to find the drift in the configuration variables and the step does not involve any evaluation of the force field defined by the potential energy function.

In the more general situation, the geodesics must be approximated by a numerical method. There are several alternative methods for computing them. The obvious approach is to employ a series of SHAKE/RATTLE steps which preserve the constraint manifold and respect the co-tangency conditions. The combined method can be viewed as a sort of multiple timestepping method in which the multiple steps are not taken with fast components of the force field but merely to resolve the geodesic flow. This approach was robust and stable in our simulations, although to push the timestep up maximally in the case of a biomolecule, we needed to employ an additional level of solvent–solute decomposition as described later in this article. Higher-order integrators [44] could be used to compute geodesics, but from our experiments, we doubt that this extra accuracy would be needed in practice in the biomolecular dynamics setting.

For the solution of $B^{g}$ we insert the equation for the acceleration into the co-tangency constraint and solve for the Lagrange multipliers. This results in a system of differential equations of the following form:

$$\begin{array}{rl} \frac{d}{dt}q & =0,\\ \frac{d}{dt}p & =-\Pi (q)\nabla U(q). \end{array}$$

The matrix ***Π*** is a projector (satisfying ***Π***^2^=***Π***) onto the cotangent space defined by

$$\Pi =\text{I}-{\text{G}}^{T}{\left[\text{G}{\text{M}}^{-1}{\text{G}}^{T}\right]}^{-1}\text{G}{\text{M}}^{-1}$$

where **G** is an *m*×*N* matrix whose rows are given by the gradients of the constraint functions, i.e.

$$\text{G}(q)=(g_{ij}(q)),\;g_{ij}(q)=\frac{\partial g_{i}(q)}{\partial q_{j}}$$

We solve the equations for ***q*** (*t*) and ***p*** (*t*) in this step of the algorithm exactly by use of the formula

q (t)≡q (0),p (t)=p (0)−tΠ(q (0))∇U(q (0))

which is a projected ‘kick’.

In the case of Langevin dynamics, the stochastic terms (due to the constrained Ornstein–Uhlenbeck process) result in a projected SDE system of the form [28,45]

3.1 $$\frac{\;dp}{\;dt}=-\gamma \Pi p+\sqrt{2\gamma k_{B}T}\Pi {\text{M}}^{1/2}\eta .$$

These equations have the (weak, i.e. exact in the sense of averages) solution

$$p\;(t)=e^{-\gamma t\Pi }p\;(0)+\sqrt{k_{B}T}{\left[\text{I}-e^{-2\gamma t\Pi }\right]}^{1/2}\Pi {\text{M}}^{1/2}R(t),$$

where ***R***(*t*)∼*N*(0,1) is a vector of i.i.d. normal random numbers.

The computation of the exponential is easily performed by Rodrigues' formula, because

$$\exp(\alpha \Pi )=\text{I}+\alpha \Pi +\frac{1}{2}\alpha^{2}\Pi^{2}+\cdots =\text{I}+\alpha \Pi +\frac{1}{2}\alpha^{2}\Pi +\cdots =\text{I}+(e^{\alpha }-1)\Pi .$$

For the square root, one thus obtains

$${\left[\text{I}-e^{-2\gamma t\Pi }\right]}^{1/2}=\sqrt{1-e^{-2\gamma t}}\Pi .$$

If we have ***Π*** ***p*** (0)=***p*** (0), then the evolution reduces to

$$p\;(t)=e^{-\gamma t}p\;(0)+\sqrt{k_{B}T(1-e^{-2\gamma t})}\Pi {\text{M}}^{1/2}R(t).$$

The detailed presentation of the g-BAOAB algorithm based on these formulas is given in the Appendix. Of course other choices of the composition sequence may be adopted. As discussed earlier in this article, we have favourable experience and supporting analysis of the g-BAOAB method in the setting of unconstrained MD [26]. We also compare the g-OBABO method in the experiments.

As the current implementation of the geodesic integrator relies on a series of SHAKE/RATTLE steps, alternatives to vanilla SHAKE, such as Newton-based techniques [14,39], LINCS [46], etc. could in principle be readily employed to implement our method.

## 4. Analysis of the sampling error

In this section, we discuss the order of accuracy of the geodesic integrator for long-term (ergodic) averages. Our approach to accuracy (in the sense of long-term averages) is at once more transparent and more relevant for MD than the standard method based on convergence on finite time intervals (‘weak order’), see, e.g. [41], which does not, in the end, typically give an asymptotic (t→∞) result because of the difficulty in computing global bounds for the error that hold on infinite time intervals.

A rigorous mathematical foundation for Langevin splitting methods for unconstrained systems, including the development of asymptotic expansions for invariant distributions and an understanding of the effect on accuracy of different ordering of the components of composition methods, is now available [36]. We summarize this approach here. The analysis proceeds from the study of the Fokker–Planck operator L acting on the phase space density. Under standard assumptions, the unique steady state of the Fokker–Planck equation ∂ρ/∂t=Lρ is the Gibbs density *ρ*_*β*_ which corresponds to thermodynamic equilibrium. The splitting of the stochastic differential equations of Langevin dynamics generates a corresponding splitting of the Fokker–Planck operator. Using the Baker–Campbell–Hausdorff expansion, one then derives an effective operator whose steady states are perturbations of *ρ*_*β*_. That is, one obtains the following relation, sometimes referred to as a Talay–Tubaro expansion [47]:

$$[L+\delta tL_{1}+O(\delta t^{2})]\;\rho_{\beta }[1+\delta tf_{1}+O(\delta t^{2})]=0,$$

where $L_{1}$ is the leading perturbation of the operator L and *f*_1_ describes the corresponding perturbation of the density. Then one obtains, after using $L\rho_{\beta }=0$, an equation for *f*_1_.

To analyse the error expansion for the invariant distribution in the constrained case, we use the method of parameterization, whereby the constrained system is replaced by an unconstrained system in a reduced coordinate system. For a general constraint manifold, this reduction can only be carried out locally and one typically needs to assemble a collection of such parameterizations to span the entire constraint manifold. However, in MD, many constraint networks we would be interested in are tree-structured [39], which implies that a single global parameterization is possible. We assume such a case here for the purpose of presenting a simplified general analysis.

After reduction to canonical coordinates ***θ***, ***p***_*θ*_, which follows by similar analysis to the deterministic case [35], the stochastic constrained system is equivalent to Langevin dynamics applied to a conservative system with a non-constant mass matrix having a Hamiltonian of the form

$$\hat{H}(\theta ,p_{\theta })=\frac{1}{2}p_{\theta }^{T}\text{J}(\theta )p_{\theta }+\hat{U}(\theta ),$$

with symmetric positive definite matrix **J**(***θ***). The Langevin equations of motion become

4.1a $$\dot{\theta }=\text{J}(\theta )p_{\theta }$$

and

4.1b $${\dot{p}}_{\theta }=-\nabla \hat{U}(\theta )-\frac{1}{2}\nabla {p_{\theta }}^{\;T}\text{J}(\theta )p_{\theta }-\gamma p_{\theta }+\sqrt{2k_{B}T\gamma {\text{J}}^{-1}(\theta )}\eta (t)$$

where ***η***(*t*) denotes a vector of Wiener increments in the appropriate dimensional space and ∇ represents the gradient with respect to ***θ***. The dynamics have an associated invariant distribution ${\hat{\rho }}_{\beta }^{g}$, where

$${\hat{\rho }}_{\beta }^{g}\propto \exp(-\beta \hat{H}(\theta ,p_{\theta })),\;\langle f(\theta ,p_{\theta })\rangle \propto \int f(\theta ,p_{\theta }){\hat{\rho }}_{\beta }^{g}(\theta ,p_{\theta })\;d\theta \;dp_{\theta }$$

Each of the substeps of the geodesic integrator, $A^{g}$, $B^{g}$ and $O^{g}$ map directly to associated parameterized integration steps ${\hat{A}}^{g}$, ${\hat{B}}^{g}$, ${\hat{O}}^{g}$:

$$\begin{array}{rl} & {\hat{A}}^{g}:\left\{ \begin{array}{rl} & \dot{\theta }=\text{J}(\theta )p_{\theta }\\ & {\dot{p}}_{\theta }=-\frac{1}{2}\nabla_{\theta }({p_{\theta }}^{\;T}\text{J}(\theta )p_{\theta }) \end{array}\right.,\;{\hat{B}}^{g}:\left\{ \begin{array}{rl} & \dot{\theta }=0\\ & {\dot{p}}_{\theta }=-\nabla \hat{U}(\theta ) \end{array}\right.\\ & {\hat{O}}^{g}:\left\{ \begin{array}{rl} & \dot{\theta }=0\\ & {\dot{p}}_{\theta }=-\gamma p_{\theta }+\sqrt{2k_{B}T\gamma {\text{J}}^{-1}(\theta )}\eta (t) \end{array}\right. \end{array}$$

This case is not directly covered by our previous convergence analysis [36], which always assumed a constant mass matrix. Nevertheless, it is possible in principle to carry out similar calculations based on the Talay–Tubaro expansion [47].

The Fokker–Planck (forward Kolmogorov) equation gives the evolution of a distribution *ρ* under the exact (weak) flow, via $\rho_{t}=\exp(tL)\rho_{0}$, where L is a second-order Kolmogorov operator. The associated evolution operator can be computed for each of the A, B and O parts:

$$\begin{array}{rl} L_{{\hat{A}}^{g}}\rho & =-\nabla_{\theta }\cdot (\text{J}(\theta )p_{\theta }\rho )+\frac{1}{2}\nabla_{p_{\theta }}\cdot (\nabla_{\theta }{p_{\theta }}^{\;T}\text{J}(\theta )p_{\theta }\rho ),\\ L_{{\hat{B}}^{g}}\rho & =\nabla_{\theta }\hat{U}(\theta )\cdot \nabla_{p_{\theta }}\rho \\ \mathrm{and}\;L_{{\hat{O}}^{g}}\rho & =\nabla_{p_{\theta }}\cdot (p_{\theta }\rho )+k_{B}T{\text{J}}^{-1}(\theta ):\nabla_{p_{\theta }}^{2}\rho \end{array}$$

where *X*:*Y* :=*tr*(*XY* ^*T*^). As we have used an additive splitting of the vector field in (4.1), the overall exact operator becomes $L_{g}=L_{{\hat{A}}^{g}}+L_{{\hat{B}}^{g}}+L_{{\hat{O}}^{g}}$ such that

$$\exp(L_{g}){\hat{\rho_{\beta }}}^{g}={\hat{\rho_{\beta }}}^{g},\;L_{g}{\hat{\rho_{\beta }}}^{g}=0.$$

For a splitting scheme composed by solving the corresponding vector fields in sequence, the overall evolution of a distribution is characterized by the product of the exponential of these operators. For example, for the g-BAOAB scheme with stepsize *δt*, we have

$$\exp(L_{g}^{\left(g\text{-}\mathrm{BAOAB}\right)}):=\exp\left(\frac{\delta t}{2}L_{{\hat{B}}^{g}}\right)\exp\left(\frac{\delta t}{2}L_{{\hat{A}}^{g}}\right)\exp(\delta tL_{{\hat{O}}^{g}})\exp\left(\frac{\delta t}{2}L_{{\hat{A}}^{g}}\right)\exp\left(\frac{\delta t}{2}L_{{\hat{B}}^{g}}\right).$$

Under an ergodicity assumption, we have a unique distribution such that

$$L_{g}^{\left(g\text{-}\mathrm{BAOAB}\right)}{\hat{\rho }}_{\beta }^{\left(g\text{-}\mathrm{BAOAB}\right)}=0,$$

with computed averages of an observable *f* (ignoring sampling error) given as

$${\left\langle \;f(\theta ,p_{\theta })\right\rangle }_{\left(g\text{-}\mathrm{BAOAB}\right)}:=\int f(\theta ,p_{\theta }){\hat{\rho_{\beta }}}^{\left(g\text{-}\mathrm{BAOAB}\right)}(\theta ,p_{\theta })\;d\theta \;dp_{\theta }.$$

If we make the ansatz that

$${\hat{\rho_{\beta }}}^{\left(g\text{-}\mathrm{BAOAB}\right)}={\hat{\rho }}_{\beta }^{g}(\theta ,p_{\theta })+\delta t\rho_{1}(\theta ,p_{\theta })+O(\delta t^{2}),$$

for some smooth perturbation *ρ*_1_, then using the Baker–Campbell–Hausdorff expansion it is useful to rewrite

$$\begin{array}{rl} L_{g}^{\left(g\text{-}\mathrm{BAOAB}\right)} & =L_{0}+\delta tL_{1}+O(\delta t^{2}),\\ L_{0} & =L_{{\hat{A}}^{g}}+L_{{\hat{B}}^{g}}+L_{{\hat{O}}^{g}}=L_{g},\\ L_{1} & =[L_{{\hat{A}}^{g}},L_{{\hat{B}}^{g}}]+[L_{{\hat{B}}^{g}},L_{{\hat{O}}^{g}}]+[L_{{\hat{O}}^{g}},L_{{\hat{A}}^{g}}]=0, \end{array}$$

by virtue of a Jacobi identity, where [*X*,*Y* ]:=*XY* −*Y* *X* is the commutator of *X* and *Y* . Thus the equation $L_{g}^{\left(g\text{-}\mathrm{BAOAB}\right)}{\hat{\rho_{\beta }}}^{\left(g\text{-}\mathrm{BAOAB}\right)}=0$ becomes

$$(L_{g}+O(\delta t^{2}))({\hat{\rho_{\beta }}}^{g}(\theta ,p_{\theta })+\delta t\rho_{1}(\theta ,p_{\theta })+O(\delta t^{2}))=0,$$

which implies that *ρ*_1_(***θ***,***p***_*θ*_)≡0 and hence

$${\left\langle \;f(\theta ,p_{\theta })\right\rangle }_{\left(g-\mathrm{BAOAB}\right)}=\langle \;f(\theta ,p_{\theta })\rangle +O(\delta t^{2}),$$

implying that we obtain at least second-order agreement with the exact result for the g-BAOAB scheme. Similar analysis holds for any scheme using this splitting such that its codifying string is a palindrome. However, while we expect a second-order error for general observable *f*, the pre-factor to the *δt*^2^ term may be very different for schemes with different compositions. In the unconstrained case, our previous work [25,36] has shown that, for particular families of observables, some higher-order terms terms can be cancelled out completely, after averaging out over the momenta. Unfortunately, preliminary calculations and numerical experiments demonstrate that such a property is lost for general symmetric positive definite **J**(***θ***); hence, the g-BAOAB scheme does not have the same superconvergence properties as in the case of constant mass matrix. Nevertheless, as numerical experiments presented below demonstrate, the error in sampling using g-BAOAB is dramatically smaller than for other geodesic splitting schemes.

## 5. Solvent–solute splitting

In our simulations of biomolecules, we use constraints in a relatively conservative way, freezing only the water molecules and the bond lengths between hydrogens and heavier atoms (N, C, O). After the introduction of constraints, we find that the dominant oscillatory mode is associated to unconstrained angle bonds in the solute molecule, which is typically a polymer or protein, thus the atoms involved in the protein model need to be simulated using smaller timesteps than the atoms in the solvent bath. This is precisely the setting that motivated the development of the RESPA (multiple timestepping) algorithm [6,7], and we turn to this device to enable a longer timestep than would otherwise be feasible.

In biomolecular simulations with detailed solvent, the number of water molecules that need to be incorporated must grow in direct proportion to the volume of the periodic simulation box, whereas even if it is in a compact state, the solute never comes close to packing the box. For an alanine dipeptide simulation (22 atoms), around 500 water molecules are typically incorporated in the simulation. For larger molecules, e.g. the bovine pancreatic trypsin inhibitor (BPTI) with 1101 atoms, the total number of atoms (14 281 in one representative simulation [48]) is again much greater because of the incorporation of detailed solvent. A simulation of mouse acetylcholinesterase [49] involved 8289 solute atoms and 75 615 solvent atoms. Thus the number of solvent–solvent interaction forces is much larger than the number of interactions between atoms of the solute and/or between atoms of the solute and those of the solvent. Thus the computational cost of computing the solvent–solvent interactions is typically the dominant cost of the timestep. While there are ways of reducing the long-ranged force computations, they remain by far the dominant cost in typical bio-MD simulation.

In our scheme, we employ RESPA in an extremely restricted form, with just two or three ‘fast’ solute steps to each outer step. In this formulation, with a complicated reduced model which is deterministically and stochastically coupled, and a small differential in timestep, we do not anticipate or observe the resonance artefact that can sometimes arise in other RESPA-based simulations [11,12]. In the fast system, we incorporate both the internal solute–solute interactions and the interactions between solute and solvent, because, on the scale of the long stepsize, the neglect of these could introduce steric clashes. In the spirit of geodesic integration, we resolve the constrained flow in tandem with the solute kicks instead of projecting only at the end of the sequence.

Let ***q***=(***q***_*p*_,***q***_*s*_) denote coordinates representing the position of the protein (or more generally, the solute) and solvent degrees of freedom, respectively, with a consequent division of the potential energy terms as

$$U(q)=U_{\mathrm{ss}}(q_{s})+U_{\mathrm{ps}}(q_{p},q_{s})+U_{\mathrm{pp}}(q_{p})$$

and a corresponding splitting of the $B^{g}$ vector field into $B_{p}^{g}$ (including forces owing to *U*_pp_ and *U*_ps_) and $B_{s}^{g}$ involving only the solvent–solvent terms, the assumption being that computation of $B_{p}^{g}$ is much cheaper than that of $B_{s}^{g}$. The evolution operator for the scheme with stepsize *δt*, using *K*_p_ intermediate solute steps, becomes

5.1 $$\begin{array}{rl} & \exp\left(\frac{\delta t}{2}L_{B_{s}^{g}}\right)(\exp\left(\frac{\delta t}{2K_{p}}L_{B_{p}^{g}}\right)\exp\left(\frac{\delta t}{2K_{p}}L_{A^{g}}\right)\exp\left(\frac{\delta t}{K_{p}}L_{O^{g}}\right)\\ & \;\;\;\times {\exp\left(\frac{\delta t}{2K_{p}}L_{A^{g}}\right)\exp\left(\frac{\delta t}{2K_{p}}L_{B_{p}^{g}}\right))}^{K_{p}}\exp\left(\frac{\delta t}{2}L_{B_{s}^{g}}\right) \end{array}$$

Analysis similar to that included above shows that we would expect this scheme to be second order. In the coordinate description, one finds that $[L_{{\hat{B}}_{s}^{g}},L_{{\hat{B}}_{p}^{g}}]=0$ and

$$\exp(\delta tL_{{\hat{B}}_{s}^{g}})\exp(\delta tL_{{\hat{B}}_{p}^{g}})=\exp(\delta tL_{{\hat{B}}_{s}^{g}}+\delta tL_{{\hat{B}}_{p}^{g}})=\exp(\delta tL_{{\hat{B}}^{g}})$$

and hence when *K*_p_=1 the scheme reduces to g-BAOAB. Note that while we would expect stability to improve as we increase *K*_p_, it is not necessarily true that the error is also reduced. In the unconstrained case, the BAOAB scheme has a special structure in its leading order error term that leads to a fortunate cancellation in the observed bias. This error term is modified with any changes in the scheme, so even attempting to increase the accuracy by solving some terms more accurately can lead, somewhat surprisingly, to an *increase* in the bias introduced to the simulation.

The stability restriction on the size of the timestep of the g-BAOAB method is a consequence of the properties of the system being simulated. In typical cases, we expect this to be a biological molecule embedded in a water bath. The spectral peaks in water due to intramolecular interactions begin around 3500 cm^−1^ (with the fastest motions related to chemical O–H, N–H and C–H bonds). These are followed by other bond stretches (e.g. the O–C–O symmetric stretch at 2400 cm^−1^), other bond stretches involving carbons, and finally angle bonds arising at around 1600 cm^−1^ [50]. The intermolecular forces yield a first peak for the out-of-plane libration at 725 cm^−1^. If these modes were purely harmonic, the first bond stretch would represent a stepsize restriction at about 3 fs, although anharmonicities alter these thresholds in numerical methods (see Schlick *et al.* [11] and Skeel and Srinivas [51]), and the incorporation of stochastic perturbations further complicates this picture. In practice, one does in fact find that the stepsize stability threshold for liquid water is limited to about 2.7 fs, or around 10% below the harmonic estimate of 3.04 fs. In our simulations, we remove the fastest bonds involving hydrogen which still leaves various solute bond stretches and angles with slightly longer periods and which normally would require stepsizes (from a harmonic estimate) of around 4.5 fs for stable integration.

These observations suggest that *K*_p_=2 or *K*_p_=3 interior steps of the constrained solute molecule are all that is needed to overcome the stability restrictions because of internal unconstrained angle bonds with an overall integration stepsize of up to the vicinity of 10 fs; in practice (see below) we found again a reduction owing to anharmonic effects, but were still able to use stepsizes of around 8–9 fs.

## 6. Application of g-BAOAB to TIP3P water

We begin with an analysis of the performance of g-BAOAB on a simple system: a box of water. For convenience, we have implemented the algorithm within the Tinker software package (http://dasher.wustl.edu/tinker/), see the electronic supplementary material for more details. We consider a constant temperature simulation of 216 TIP3P [52] water molecules in a 18.643 Åperiodic box, included as one of the Tinker benchmark simulations. We run at 300K using a 9 Å cutoff for all interactions, using constraints on both angles and bond lengths making each water molecule completely rigid. We compared the following:

— the Midpoint–Euler–Verlet–Midpoint–Euler (MEVME) method of Lelievre *et al.* [28],
— the scheme implemented in the Tinker standard package (v 7.1) and referred to there as the Velocity Verlet stochastic dynamics method (derived from a method of Guarneri and Still [53]), and run using the command *stochastic*,
— geodesic versions of the Bussi–Parinello method [24], denoted g-OBABO,
— the geodesic BAOAB (g-BAOAB) method.

We further compare using either one or five steps of the RATTLE algorithm for each of the A pieces in the g-OBABO and g-BAOAB schemes.

**Figure 2.**
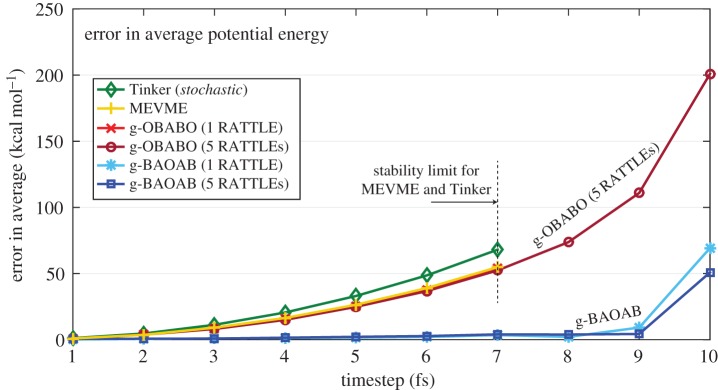
The error in average total potential energy for the TIP3P model system is plotted for each scheme in comparison to the baseline result of − 2078 *kcal* *mol*^−1^. The MEVME and Tinker default schemes were unable to complete a simulation past a timestep of 7 fs (and then with high error), whereas the g-BAOAB scheme gives a very small error in total potential energy up to 9 fs. Using more accurate geodesics improves the stability of g-OBABO, but with relatively large error.

We run simulations using each scheme with a timestep of between 1 and 10 fs, over a fixed time period of 5 ns and with the friction parameter set to a standard value 1 *ps*^−1^. For each experiment, results are averaged over five independent runs. Computed results are compared to the average of five baseline simulations computed using the default Tinker integrator with a timestep of 0.5 fs. In figure 2 we show how the average total potential energy varies as we change the timestep for each scheme.

It is clear from the figure that the g-BAOAB scheme is far superior for the computation of configurational quantities such as average potential energy compared to the other schemes. When using one RATTLE step per A piece (each RATTLE iteration increased wall clock time by 4% in our simulations) the timestep for the g-BAOAB scheme can be pushed as far as 9 fs for TIP3P without significant error. The observed error at 9 fs is comparable to the error in standard schemes at between 2 and 3 fs. Increasing the number of RATTLE steps does not provide a significant qualitative difference to the behaviour of the g-BAOAB scheme, since the sampling error is too large to further resolve the precision of the integrator and because the baseline result is computed using the Tinker default integrator which introduces substantial bias.

Interestingly, in the g-OBABO scheme, increasing the number of RATTLE steps taken improves the stability of the scheme but has little effect on the error. One would expect that converting the drift steps in the Tinker or MEVME schemes to a geodesic update using multiple RATTLE steps would improve the stability of the schemes beyond 7 fs, but likely not improve the existing error results for smaller timesteps.

**Figure 3.**
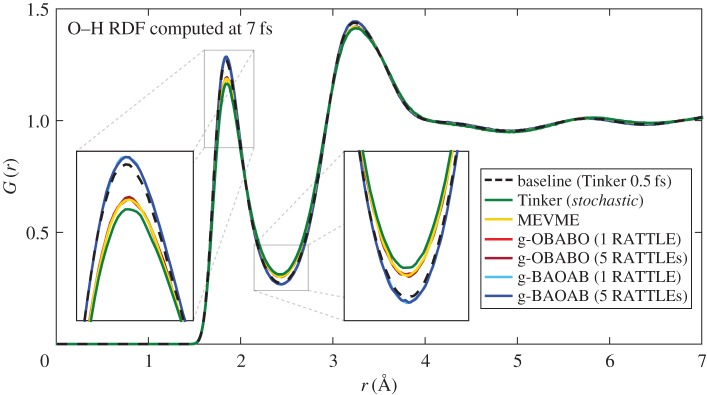
Computed O–H radial distribution functions *G*(*r*) for each scheme at a timestep of 7 fs, compared to the baseline result computed at 0.5 fs (black, dashed). The initial peak and trough are highlighted in the insets.

 Figure 3 gives the computed radial distribution function for the O–H distance in the simulation, using the Tinker package's *radial* analysis program. We plot the curves for simulations using a timestep of 7 fs, and compare to the baseline result at 0.5 fs.

Although errors are present in all schemes, it is again the g-BAOAB scheme which gives the best results. The other schemes appear to heat the system artificially, pulling the *G*(*r*) curve closer to unity in the initial peak and trough. We can verify this effect by looking at the diffusion coefficient averaged over all repeated experiments, given in table 1.

**Table 1.** Computed diffusion coefficient of water (×10^−5^ cm^2^ s^−1^).

Scheme	3 fs	5 fs	7 fs	9 fs
Tinker default	4.59 ± 0.13	5.08 ± 0.37	5.81 ± 0.37	fail
MEVME	4.73 ± 0.30	4.85 ± 0.23	5.69 ± 0.34	fail
g-OBABO (1 RATTLE)	4.65 ± 0.18	5.33 ± 0.42	5.69 ± 0.34	fail
g-OBABO (5 RATTLEs)	4.79 ± 0.31	5.14 ± 0.38	5.40 ± 0.40	6.39 ± 0.15
g-BAOAB (1 RATTLE)	4.48 ± 0.32	4.41 ± 0.19	4.54 ± 0.26	4.61 ± 0.31
g-BAOAB (5 RATTLEs)	4.52 ± 0.34	4.51 ± 0.20	4.67 ± 0.45	4.65 ± 0.30

In assessing the accuracy of diffusion rates, we are faced with several issues. First, it is well known that the TIP3P model does not accurately model the self-diffusion of water [54], with an observed rate of 5.06×10^−5^ cm^2^ s^−1^, around double the realistic value. In addition, Langevin dynamics reduces the diffusion compared to microcanonical calculation by an amount dependent on the magnitude of the friction coefficient [17]. Our comparisons are therefore only sensible relative to an accurate discretization of Langevin dynamics with the same friction coefficient, in this case *γ*=1 ps^−1^. (If the key purpose of the simulation is highly accurate intermediate time dynamics, then one should either resort to a very small friction, use microcanonical simulation or else employ an alternative ‘gentle’ thermostat [55].)

The diffusion constant is approximated using Tinker's *diffuse* program, which computes mean squared displacements from snapshots taken every 1 ps for each trajectory. Our baseline computation gives the mean diffusion constant as 4.515×10^−5^ cm^2^ s^−1^, with an error of 0.186×10^−5^ cm^2^ s^−1^. All the schemes give consistent results for the diffusion coefficient at a 3 fs timestep, and the g-BAOAB scheme gives reasonably accurate results using a 9 fs timestep, even when using a single RATTLE step. However, there is evident bias introduced in the other schemes, with the increased diffusion suggesting a higher temperature being sampled than that prescribed by the thermostat.

In this example, each additional RATTLE step increased the wall clock time by approximately 4%, making the g-BAOAB scheme run using five RATTLE iterations take about 36% longer compared to the standard Tinker integrator. Thus, on the basis of efficiency measured as accuracy per unit computational work, the optimal method in this case involves just a single RATTLE step in each $A^{g}$ solve. With a single RATTLE step the overall wall clock time is similar for all of the popular methods tested.

We note that the case of independent rigid bodies coupled through forces, such as the TIP3P model described in this section, can also be handled using the quaternionic Langevin schemes of Davidchack *et al.* [43]. One of the methods (‘Langevin C’) of that article uses a BAOAB-inspired scheme in quaternionic representation; the authors of that article likewise observed a maximum stepsize of around 9 fs with high configurational sampling accuracy, suggesting a close relationship between our scheme and theirs in this special case. However, their method does not extend to more complicated constraint manifold structures such as those associated to a protein.

## 7. Application to a biomolecular model problem

We consider a simulation of solvated alanine dipeptide, parameterized using the Amber99 [56] force field with explicit solvent and an 8 Åcutoff for all long-range interactions. The NVT simulation was conducted at 300 K in a 24 Å periodic cube, with 442 TIP3P water molecules kept rigid and with all bonds to hydrogen in the alanine dipeptide molecule constrained.

Testing showed that the solvent–solvent interactions were by far the most costly computation in the simulation, taking roughly 95% of the total force-call time. Hence we expect that performing the multiple timestep version of g-BAOAB (based on a solvent–solute splitting) will in practice be only moderately more costly than a standard constrained Langevin scheme, given an efficient implementation. (Additional discussion of efficiency is taken up in the conclusion.)

**Figure 4.**
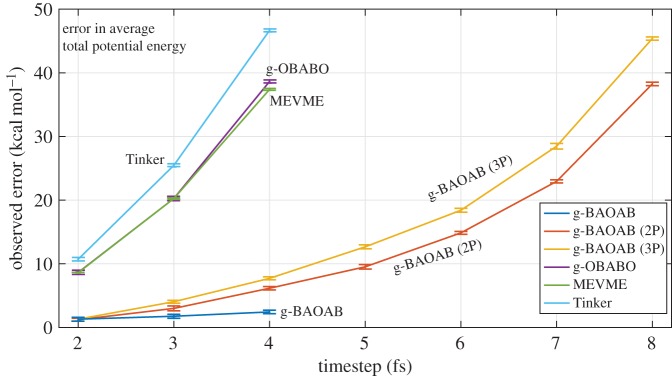
The error in the observed average potential energy for solvated alanine dipeptide, computed at timesteps of between 2 and 8 fs. Results are averaged over ten 5 ns trajectories and are to be compared to a baseline result of −4240 *kcal* *mol*^−1^ for the average total potential energy. Using additional protein force evaluations for the g-BAOAB scheme (the value of *K*_p_ used in (5.1) is given in brackets) we are able to significantly increase the timestep.

Results are computed from 10 independent trajectories of fixed time 5 ns, using a friction of 0.5 ps^−1^ and using one RATTLE step for the A update in g-OBABO and g-BAOAB. In figure 4 we plot the error in the average potential energy observed in the simulation, when compared to a baseline result computed from twenty trajectories using the Tinker thermostat at a timestep of 0.5 fs.

All of the standard Langevin schemes (without multiple force calls) became unstable beyond a timestep of 4 fs; however, the g-BAOAB scheme demonstrates an order of magnitude improvement in the observed error in the scheme. It is notable that even when run at 4 fs, the g-BAOAB scheme is still over four times more accurate than conventional schemes run at half the timestep. However, increasing the number of solute calls evidently doubles the stability of the schemes, with a modest impact on the wall clock time in an efficient implementation.

When using three protein steps per timestep we were able to run stable simulations at stepsizes of up to 10 fs. As this was the limit of stability for the solvent-only case (demonstrated in §6), we would not expect any further stability increase beyond this point. The scheme also made larger errors in average potential energy for runs with stepsize above 8 fs. The mean observed error at a stepsize of 9 fs was 102 kcal mol^−1^ (against a total energy of around −4240 kcal mol^−1^).

An unexpected result is that using more solute steps per timestep appears to slightly increase the reported error in average potential energy. As we are effectively using a smaller timestep for the solute dynamics, it would be reasonable to expect an improvement in overall accuracy compared to the more general g-BAOAB scheme. However, this result can be explained given the framework we have developed. Using more solute steps alters the sequence of Fokker–Planck operators that characterize the evolution of measure for a particular scheme. In the unconstrained dynamics, the BAOAB scheme is a special case of the five-term palindrome sequences that can be shown to exhibit special cancellations that reduce the total observed error. Changing the method alters the resulting propagation of distribution and appears to destroy the structure inherent in the operator expansion that allows these cancellations.

**Figure 5.**
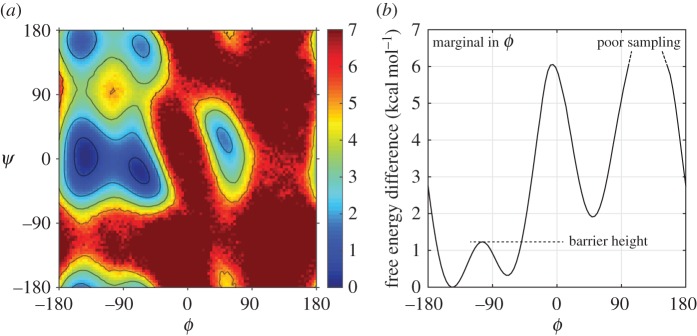
(*a*) The free energy plot in the (*ϕ*,*ψ*) dihedral angle space, computed from a baseline result. Contours of free energy are plotted at 0.5, 2, 3.5 and 5 kcal mol^−1^. (*b*) The marginal distribution is plotted for the *ϕ* dihedral angle. The barrier height estimated in figure 7 is marked with the dash line.

**Figure 6.**
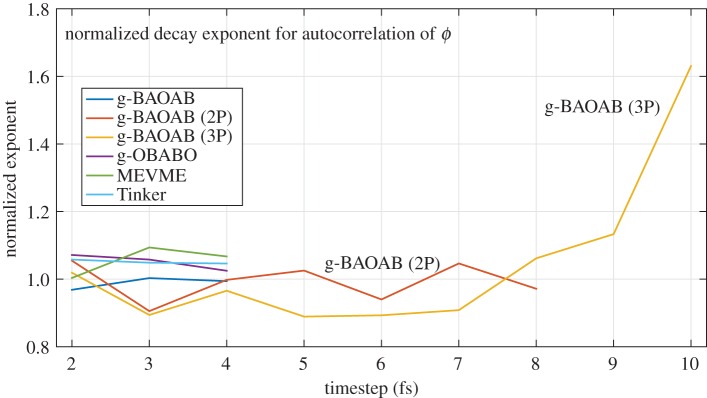
The decay of the autocorrelation function of an indicator on the *ϕ* variable is estimated by fitting the computed curve to an exponential, and then normalized based on a baseline run. The g-BAOAB scheme, in each of the variants, gives an accurate result between 0.9 and 1.1 until 8 fs.

We also compare the rate of decay of the autocorrelation function for an indicator function of the *ϕ* dihedral angle of the alanine dipeptide protein. We use the indicator function *f*(*ϕ*) where

$$f(\phi )=\left\{ \begin{array}{ll} 1 & -100<\phi <-20\\ 0 & \mathrm{otherwise} \end{array}\right.$$

and compute its autocorrelation function from sixteen 5 ns trajectories, generated using the standard Tinker scheme at 1 fs. This indicator approximately marks a region covering one free energy well in the *ϕ* coordinate, as is shown in figure 5. In an effort to reduce the overall sampling error, we fit the averaged curves for each scheme to a decaying exponential function (using MATLAB's *fit* function) and compare the resulting exponents. The normalized exponents are plotted in figure 6.

Despite the noise in computing the autocorrelation function, the g-BAOAB method (both variants) gives agreement in the integrated autocorrelation time (within sampling error) up to around 8 fs. With three solute steps, the method can be pushed to 9 fs, while at 10 fs a significantly poorer result is obtained, with diffusion happening much faster than expected. This is likely due to the effect of bias introduced into the distribution lowering the free energy barriers present in the system, thus changing the timescales observed in barrier crossings.

**Figure 7.**
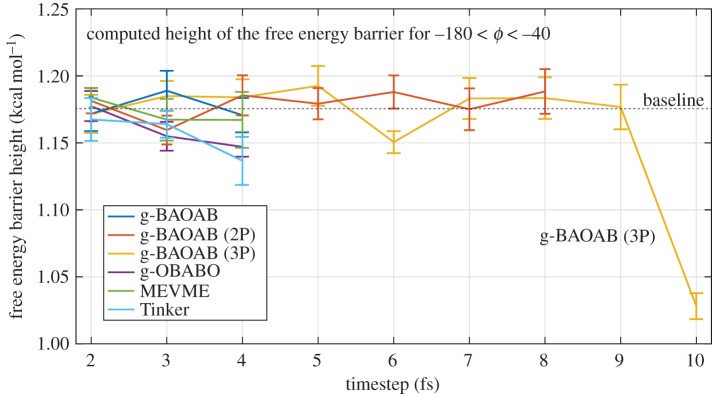
The estimated free energy barrier height in the *ϕ* coordinate is plotted as the stepsize is varied for each scheme. The g-BAOAB method is stable and accurate to stepsizes above 8 fs, giving a substantially correct effective free energy barrier height.

To show this effect, we estimate the height of the free energy barrier (marked in the right plot of figure 5) for −180<*ϕ*<−40. We plot the results in figure 7, with the baseline result of 1.76±0.01 kcal mol^−1^ denoted with a dashed line. We recover a good estimate for the g-BAOAB method with stepsizes up to around 9 fs. In contrast, for example, the Tinker scheme performs poorly at the maximum 4 fs stepsize, with an error beyond 10%, contributing to an increased diffusion across the barrier.

In this example the constraint topology of the solute molecule is relatively simple, consisting of a number of small decoupled, tree-structured groups of atoms. The constraints are therefore quite easy to maintain and it was only necessary to use a single RATTLE step in each A piece to maintain good stability. We expect that if more bonds (or bonds and angles) are constrained, then the resolution of the constraint geometry will become more delicate and likely necessitate a more accurate approximation of the geodesics.

## 8. Conclusion

The geodesic BAOAB method offers both accuracy and stability enhancements for the biomolecule in atomistic solvent. The accuracy improvement due to the use of the g-BAOAB method (without the solvent–solute splitting) at a 4 fs timestep, the largest stepsize usable by most standard constraint algorithms, is around one order of magnitude for configurational averaging (see figure 4). This is consistent with our experience of the corresponding unconstrained scheme [26] and indicates that the g-BAOAB method is the proper analogue of BAOAB for constrained systems.

When simulations are performed in a pre-converged setting (in which sampling error, rather than perfect sampling bias, is the main obstacle) the stability rather than the accuracy of the method becomes central. By combining g-BAOAB with a multiple timestepping procedure, the constrained bio-MD stability threshold can be reliably lifted to around 8 fs. Implementing the large timesteps efficiently requires separation of solvent and solute force computations and the efficient treatment of neighbour lists. In our unoptimized Tinker implementation, we found that the cost of a full timestep using our 8 fs (with two solute steps) g-BAOAB method was about 20% greater than a 4 fs step of the standard Tinker scheme. However, we attribute much of the difference to computational work in the updating of neighbour lists (Tinker performed this operation for the full system at each intermediate force call, regardless of which atoms actually needed updating) and the small size of the alanine dipeptide system. In larger systems and with moderate optimization of the force calculation, we would expect the overhead to be no greater than around 10%. On the other hand, careful attention to some of the figures of the last section (see, e.g. figure 7) suggests that the stepsize for the standard algorithms should be limited to less than around 3 fs whereas g-BAOAB appears to be robust at 8 fs, so the net efficiency gain is likely to be more than 100%.

The scheme proposed here incorporates three separate mechanisms: (i) the more accurate approximation of geodesics, (ii) the use of a solvent–solute force decomposition in RESPA-style multiple timestepping, and (iii) the fortuitous cancellation of errors in the invariant distribution because of the specific ordering of the component parts (BAOAB). It is difficult to completely separate the roles of each aspect. For example the constraints needed to be resolved once per solute step, so they are in effect more accurately treated at the large stepsize whenever solvent–solute splitting is used. We conjecture that many other integrators could be stabilized by the geodesic integration procedure and the use of solvent–solute splitting, but this only makes sense provided the sampling bias due to discretization error can be controlled. For example, using more accurate resolution of the geodesics (five RATTLE steps) improved the stability of the g-OBABO method for TIP3P water, allowing stepsizes to be increased substantially, but with much higher sampling bias than for g-BAOAB.

Currently there is demand for algorithms that scale to much larger molecular sizes. In the case of a biomolecule in solvent, this involves more efficient treatment of the long range electrostatic forces, typically using Particle-Mesh Ewald and domain decomposition techniques. Restrictions in the way this is implemented within the Tinker package prevented us from performing simulations using Ewald summation within the solvent–solute splitting. Ewald summation leaves the essential solvent–solute dynamics in the vicinity of the biomolecule to be handled in a detailed atomistic model (i.e. our solvent–solute and geodesic integration methods would still certainly apply to this part). Regardless of the methods used, the solvent-related interactions remain the dominant part of a typical biomolecular simulation.

A natural question raised by the current work is where the next barriers lie. Our simulations constrained only the bond stretches involving hydrogen atoms. It is likely that the stepsize could be further increased (to 10 fs or higher) by incorporating additional constraints on all bond stretches or even angle bonds, although there is a risk that these may in some cases alter potentially important dynamical processes. There is nothing to prevent our technique from being combined with a coloured noise thermostat [57] and other improvements [18,58] to increase the stepsize further, albeit at the cost of additional algorithmic complexity, however we note that those methods generally encounter stability thresholds above about 12 fs due to the out of plane libration mode and other intermolecular interactions, so breaking this barrier would likely require significantly changes in methodology. It is unclear whether the substantial additional complexity of designing a method to bridge to 12 fs would be justified by the relatively slender efficiency gains.

Langevin dynamics is not the only possible choice for thermostatted MD. A recent article of Peters *et al.* [45] addressed some other viable canonical sampling methods, including ‘impulsive’ methods which randomize selected velocities, as well as DPD-like thermostatting schemes, also in the presence of constraints. Another family of approaches is based on ‘degenerate thermostats’ which have a relatively mild perturbative effect on dynamical properties [59,55]. Since our methods are modular and based on easily available functionalities they can also be combined with these alternative thermostats.

The methods presented here are designed to be easy to implement in standard molecular software packages. Implementation is currently being performed by the authors in the LAMMPS software package (http://www.lammps.sandia.gov). Moreover, the g-BAOAB scheme is also currently being implemented in the Molecular Integrator Software Tools package of the ExTASY project (http://www.extasy-project.org). Using MIST, the g-BAOAB algorithm will be available in conjunction with AMBER (http://www.ambermd.org) and Gromacs (http://www.gromacs.org) (with support planned for many other codes). A separate NAMD (http://www.ks.uiuc.edu/Research/namd/) implementation is in progress.

## Supplementary Material

Codes and implementation details

## Data accessibility

The Tinker code used for these experiments is available at http://datashare.is.ed.ac.uk/handle/10283/1952, see the supplementary information for details.

## Appendix

In order to aid implementation we write the algorithms using *in situ* substitution. We write ***F***(***q***)=−∇*U*(***q***) for the vector of forces. The constraint functions are *g*_*j*_, *j*=1,2,…,*m* and we write ***λ*** or ***μ*** for a vector of *m* Lagrange multipliers used to satisfy either the constraints or the co-tangency conditions. ***G***_*j*_=∇*g*_*j*_(***q***) is the normal vector to the *j*th constraint surface at the point ***q***. We introduce useful constants $a_{j}=\exp(-j\gamma \delta t/2)$ and $b_{j}=\sqrt{k_{B}T(1-a_{j}^{2})}$, and denote vectors of i.i.d. normal random numbers as ***R***. Where more than one vector of random numbers ***R*** is required per step, the vectors are always uncorrelated.

The methods all rely on projections onto the manifold M, defined by the constraints

$$q\in M\Leftrightarrow g_{j}(q)=0,\;j=1,2,\ldots ,m,$$

and/or the co-tangent space $T_{q}^{*}M$ defined the corresponding linear constraints on ***p***:

$$p\in T_{q}^{*}M\;\Leftrightarrow \;G_{j}{\left(q\right)}^{T}{\text{M}}^{-1}p=0,\;j=1,2,\ldots ,m.$$

In order to integrate the geodesic drift step, we use *K*_*r*_ iterations of the RATTLE integrator without the force term, the combination of SHAKE and RATTLE projections, a functionality available in many major codes. We write the function *A*(⋅) which performs this step in detail below:

function (Q,P):=A(q,p,δt):sp|p∗←p+∑jλjGj(q )Q←q+δtM−1p∗∈Mwhere λ is chosen so that Q∈M, i.e. so that gj(Q)=0,j=1,2,…,m,P←p∗+∑jμjGj(Q)∈TQ∗Mwhere μ is chosen so that P∈TQ∗M, i.e. so that Gj(Q)TM−1P=0,j=1,2,…,mreturn (Q,P )end

A link to download the Tinker code used in this article is given in the supplementary material.

**g-BAOAB**

p←p+δt2F(q )+∑jμjGj(q )∈Tq∗M,For k from 1 to Kr do:spa(q,p )←A(q,p,δt2Kr)end dop←a2p+b2M1/2R+∑jμjGj(q )∈Tq∗M,For k from 1 to Kr do:spa(q,p )←A(q,p,δt2Kr)end dop←p+δt2F(q )+∑jμj∇gj(q )∈Tq∗M,

**g-BAOAB with solvent–solute splitting**

We write the force as ***F***(***q***)=***F***_p_(***q***)+***F***_*s*_(***q***), where ***F***_*s*_ denotes the force from all solvent–solvent interactions, and ***F***_p_ the remaining solute–solute and solvent–solute interactions. Each iteration we use *K*_p_>0 evaluations of ***F***_p_, where choosing *K*_p_=1 reduces this method to the g-BAOAB scheme written above.

p←p+δt2Fs(q )+∑jμjGj(q )∈Tq∗M,For j from 1 to Kp do:p←p+δt2KpFp(q )+∑jμjGj(q )∈Tq∗M,For k from 1 to Kr do:spa(q,p )←A(q,p,δt2KrKp)end dop←a2/Kpp+b2/KpM1/2R+∑jμjGj(q )∈Tq∗M,For k from 1 to Kr do:spa(q,p )←A(q,p,δt2KrKp)end dop←p+δt2KpFp(q )+∑jμjGj(q )∈Tq∗M,end dop←p+δt2Fs(q )+∑jμjGj(q )∈Tq∗M,

**g-OBABO**

Note that this scheme could also be implemented in conjunction with solvent-solute splitting, if desired, in a similar fashion as in the previous algorithm.

p←a1p+b1M1/2R1+∑jμjGj(q )∈Tq∗M,p←p+δt2F(q)+∑jμjGj(q )∈Tq∗M,For k from 1 to Kr do:spa(q,p )←A(q,p,δtKr)end dop←p+δt2F(q )+∑jμjGj(q )∈Tq∗M,p←a1p+b1M1/2R2+∑jμjGj(q )∈Tq∗M,

**MEVME**

This is the Midpoint Euler–Verlet–Midpoint Euler algorithm as described in [28].

$$\begin{array}{rl} & \begin{array}{l} p\leftarrow {\left(1+\frac{\delta t}{4}\gamma \right)}^{-1}\left(\left(1-\frac{\delta t}{4}\gamma \right)p+\sqrt{\frac{\delta t\gamma }{\beta }}{\text{M}}^{1/2}R_{1}\right)+\sum_{j}\mu_{j}G_{j}(q)\in T_{q}^{*}M, \end{array}\\ & \begin{array}{l} p\leftarrow p+\frac{\delta t}{2}F(q\;)+\sum_{j}\lambda_{j}G_{j}(q),\\ q\leftarrow q+\delta t{\text{M}}^{-1}p\in M, \end{array}\\ & \begin{array}{l} p\leftarrow p+\frac{\delta t}{2}F(q\;)+\sum_{j}\mu_{j}G_{j}(q)\in T_{q}^{*}M, \end{array}\\ & \begin{array}{l} p\leftarrow {\left(1+\frac{\delta t}{4}\gamma \right)}^{-1}\left(\left(1-\frac{\delta t}{4}\gamma \right)p+\sqrt{\frac{\delta t\gamma }{\beta }}{\text{M}}^{1/2}R_{2}\right)+\sum_{j}\mu_{j}G_{j}(q)\in T_{q}^{*}M. \end{array} \end{array}$$

**Tinker default**

This scheme is used when the integrator flag is set to *stochastic* in the Tinker package (http://dasher.wustl.edu/tinker/). It is a modification of the algorithm found in [53], with appropriate constants

$$\begin{array}{rl} \kappa_{1} & =\frac{1-a_{2}}{\gamma },\;\kappa_{2}=\frac{\delta t-\kappa_{1}}{\gamma },\;\kappa_{3}=\frac{\sqrt{k_{B}T\kappa_{5}}}{\gamma },\;\kappa_{4}=\frac{{\left(1-a_{2}\right)}^{2}}{\sqrt{\kappa_{5}(1-a_{4})}},\\ \kappa_{5} & =2\gamma \delta t-3+a_{2}(4-a_{2}). \end{array}$$

Note that for small values of *γδt* care must be taken in evaluating some expressions for the *κ*_*i*_ to ensure numerical stability.

$$\begin{array}{rl} & \begin{array}{l} p_{\mathrm{old}}\leftarrow p\\ p\leftarrow a_{2}p+\frac{1}{2}\kappa_{1}F(q)\\ q\leftarrow q+\kappa_{1}{\text{M}}^{-1}p_{\mathrm{old}}+\kappa_{2}{\text{M}}^{-1}F(q)+\kappa_{3}{\text{M}}^{-1}R_{1} \end{array}\\ & \begin{array}{l} p\leftarrow p+\sum_{j}\lambda_{j}G_{j}(q),\\ q\leftarrow q+\delta t{\text{M}}^{-1}\sum_{j}\lambda_{j}G_{j}(q)\in M, \end{array}\\ & \begin{array}{l} p\leftarrow p+\frac{1}{2}\kappa_{1}F(q)+b_{2}\left(\kappa_{4}R_{1}+\sqrt{1-\kappa_{4}^{2}}R_{2}\right)+\sum_{j}\mu_{j}G_{j}(q)\in T_{q}^{*}M, \end{array}. \end{array}$$
