# Supplementary material for: Efficient molecular dynamics using geodesic integration and solvent–solute splitting
Source: Proc Math Phys Eng Sci. 2016 May;472(2189):20160138. doi: 10.1098/rspa.2016.0138 (PMC4893190; doi:10.1098/rspa.2016.0138)
Supplement: Codes and implementation details [file rspa20160138supp1.pdf]

Supplementary Material to accompany "Efficient molecular dynamics using geodesic integration and solvent-solute splitting": Codes and Implementation Details

Benedict Leimkuhler and Charles Matthews

April 5, 2016

The results reported in this article consist of molecular dynamics simulations performed using the Tinker software package (v7.1) provided free of charge at `http://dasher.wustl.edu/tinker/`. We are proposing that the g-BAOAB integration routine be incorporated in the next full release of Tinker.

A folder of auxiliary fortran routines to be used in conjunction with Tinker is provided in the University of Edinburgh Data Share facility `http://datashare.is.ed.ac.uk/handle/10283/1932`.

The specific routines provided are as follows:

baoab.f: Source code for the g-BAOAB langevin integrator.

baoabmts.f: Source code for the g-BAOAB integration method with solvent-solute splitting.

mevme.f: Source code for the MEVME routine of Lelievre, Rousset and Stoltz, as mentioned in the article.

obabo.f: Source code for the g-OBABO langevin integrator.

In order to use these routines, it will be necessary to modify Tinker routines **dynamic.f** and **mdinit.f** to allow you to use the integrator's keywords (baoab, baoabmts, mevme, obabo) in the Tinker .key file.

The additional parameters relevant for the routines are NRATTLE (indicating the number of RATTLE substeps to be performed to approximate the geodesic flow) and NMTS (the number of interior solute steps to be taken at each step, either 2 or 3).

Initialization files (.parm, .key, .xyz, .dyn) for the simulations with water and with solvated alanine dipeptide are also included in the provided software repository mentioned above.

Please do not hesitate to contact the authors for assistance.
